# Cyberviolence Against Women and Girls in Spanish Adolescents: Experiences of Cyberaggression and Cybervictimization

**DOI:** 10.3390/bs15091165

**Published:** 2025-08-26

**Authors:** Virginia Ferreiro Basurto, Esperanza Bosch Fiol, Maria Antonia Manassero Mas, Victoria A. Ferrer-Pérez

**Affiliations:** Department of Psychology, Universitat de les Illes Balears, 07122 Palma, Spain; esperanza.bosch@uib.es (E.B.F.); ma.manassero@uib.es (M.A.M.M.); victoria.ferrer@uib.es (V.A.F.-P.)

**Keywords:** cyberviolence against women and girls, cyber behavior, adolescence, cyberaggression and cybervictimization

## Abstract

Understanding the scope of cyberviolence against women and girls in adolescents and the differences between girls and boys is a fundamental starting point for its prevention. This study analyzes the experiences of cyberaggression and cybervictimization perpetrated and suffered by 762 adolescents (399 girls and 363 boys) aged 14 and 15 in the Balearic Islands (Spain) through a diagnostic study of an electronic survey administering the Gender Violence 2.0 questionnaire. The descriptive results show that, in general, the majority of boys and girls do not commit or suffer from sexist behaviors in digital environments. A crosstab analysis (*p* < 0.001) confirms that, as expected, girls commit less cyberaggression and suffer more cybervictimization, while boys were more often the cyberaggressors and less frequently the victims. Specifically, boys claim to be cyberaggressors more often than girls, especially in relation to cybervictimization associated with sexual violence, impositions of beauty standards, and anti-patriarchal manifestations; girls claim to be cybervictims more often than boys, primarily experiencing cyberviolence related to partner cyber control and beauty standards. These results reinforce the need to design differentiated programs for the prevention of this cyberviolence: for boys, it should be focused on the cyberaggression committed, and for girls, it should be focused on identifying and coping with cyberaggression received.

## 1. Introduction

There is a growing international consensus that recognizes cyberviolence against women and girl as an alarming and expanding manifestation of gender-based violence, posing a significant threat to public health and constituting a serious violation of human rights. In this context, various international institutions have begun to conceptualize and define this phenomenon with increasing precision. The glossary of the European Institute for Gender Equality (EIGE, 2017a) defines cyberviolence against women and girls (CVAWG) as “gender-based violence that is perpetrated through electronic communication and the internet”. Additionally, the EIGE (2017b) reminds us that cyberviolence is not a separate phenomenon from ‘real-world’ violence, as it often follows the same patterns as offline violence and also that although cyberviolence can affect both women and men, women and girls experience different and more traumatic forms of cyberviolence.

The CEDAW General Recommendation No. 35 on gender-based violence against women (UN, 2017) consider that the term “gender-based violence against women” is more precise than speaking only about “violence against women” because it makes explicit the gendered causes and impacts of the violence. It point outs that this violence occurs in all spaces and spheres of human interaction, whether public or private, including online and in other digital environments.

GREVIO’s General Recommendation No. 1 (GREVIO, 2021) underscores that the digital dimension of violence against women encompasses a wide range of harmful behaviors perpetrated online or through the use of technologies (activities conducted using technological and communication equipment, including hardware and software). These cyber behaviors form part of the continuum of violence perpetrated against women and girls, which is rooted in the same context of women’s inequality and men’s sense of entitlement as the psychological, sexual and physical violence experienced by women and girls in the offline world.

And in Spain, the State Strategy to Combat Gender-Based Violence 2022–2025 (DGVG, 022) refers to this form of violence as violence exerted or facilitated through technology.

In this paper, we will use the term CVAWG as it encompasses a greater number of forms of cyberviolence and, at the same time, explicit references to a form of gender-based violence. In addition, the term cyber behavior will be used to refer to the specific behaviors occurring in digital environments that are analyzed in this study, which was conducted with Spanish adolescents.

Once the key concepts under investigation have been clarified, it is essential to analyze their prevalence and understand to what extent this problem affects Spanish adolescents. This is particularly relevant given the growing body of research highlighting the increasing influence of cyberviolence in daily life, especially among women and adolescents.

Recent years have seen a considerable rise in these technology-facilitated violence or cyberviolence, with a notably high impact on women. For example, a study conducted by the Pew Research Center (Vogels, 2021) that analyzes different forms of CVAWG found that 75% of the 10,000 participants surveyed had suffered some type of cyberviolence on social networks, with a greater incidence among women than among men by 13 percentage points. The CVAWG normalizes sexist habits and routines, that reproduce and reinforce gender stereotypes (EIGE, 2017b) in digital environments, intensifying news manifestations of violence perpetrated against women, such as cyber intimate partners violence or sexual violence (Council of Europe, 2019).

We can affirm that violence against women and girls is a public health crisis (UN, 2021), aggravated by the incorporation of new technologies. And, as the General Recommendation No. 19 (CEDAW, 2017) recognizes, technology-facilitated violence against women constitutes a new and contemporary form of virtual violence.

Meanwhile, social network platforms have become the primary venue not only for social interaction among younger generations (EIGE, 2017b), but also for the greatest incidences of CVAWG (Donoso-Vázquez et al., 2017, 2018; Esteban & Gómez, 2022; Ferreiro, 2024; Larranga-Paul et al., 2022; Mena-Rodríguez et al., 2022; Stonard, 2020; Velasco et al., 2018; Vilà et al., 2015; Villar et al., 2021). The Digital 2023 Global Overview Report (We Are Social, 2023) considers 12% of the social network audience to be composed of minors aged 13–19 years.

It is important to consider that social media plays a central role during adolescence, serving as a key environment for social interaction and identity development. As noted by Fabris et al. (2024), adolescents often turn to digital platforms to meet essential emotional and relational needs, including peer acceptance, identity construction, and a sense of group belonging, particularly when these needs are unmet in offline settings such as school. These authors suggest that social media can offer opportunities to maintain interpersonal connections, share experiences, and foster feelings of inclusion. However, an exclusive reliance on digital contexts to fulfill such needs may increase adolescents’ vulnerability, potentially resulting in higher levels of peer victimization, engagement in aggressive behavior, and psychosocial maladjustment (Martínez-Ferrer et al., 2018). This highlights the ambivalent nature of these environments, which may function both as a source of support and as a setting for psychological distress, behavioral risks, and diminished aca-demic performance if not adequately managed (Fabris et al., 2024).

Overall, active participation in the digital world at such an early age exposes adolescents to a greater risk of experiencing cyberviolence at an early age. In fact, there is a considerable presence of young people from the age of 12 in social networks, 75.1% of Europeans have suffered some type of cyberviolence in their childhood (Del Moral & Sanjuán, 2019), and the first occurrence of victimization taking place is between the ages of 14–15 (International Plan, 2020). Moreover, young girls are much more likely to experience cases of this cyberviolence, and, particularly, cases of CVAWG, such as cyber controlling behavior, cyber intimate partner violence against women, or cyber sexual violence (Agencia de los Derechos Fundamentales de la Unión Europea, 2014; International Plan, 2020). The moment when girls transgress the fringe of patriarchal norms, they become relegated to a position of vulnerability and, consequently, cybervictimization (Donoso-Vázquez et al., 2014; Martínez-Ferrer et al., 2018). In addition to this, when we add the aspects related to the development of maturity inherent to this age, including low frustration tolerance, heightened impulsivity, a proclivity for reckless cyber behaviors, and increased emotional vulnerability in digital interactions (Martín-Martín et al., 2021), and the reality of facing an individual and collective social health issue, such as CVAWG.

Given their high prevalence, several studies carried out with adolescents have addressed CVAWG behaviors (Backe et al., 2018; Fernet et al., 2019). To contextualize our research, we will focus our attention on studies carried out with adolescents in Spain (Donoso-Vázquez et al., 2014, 2018; Esteban & Gómez, 2022; Mena-Rodríguez et al., 2022; Mena-Rodríguez & Velasco-Martínez, 2017; Velasco et al., 2018; Vilà et al., 2018).

In general, the findings of these studies indicate that adolescents (neither male nor female) not consider themselves as cyberaggressors or cybervictims. However, a marked trend was noted among boys perceiving themselves as more cyberaggressive (Bonilla et al., 2017; Donoso-Vázquez et al., 2016, 2017, 2018; Esteban & Gómez, 2022; Vilà et al., 2018; Villar et al., 2021) and among girls perceiving themselves as more cybervictims of CVAWG (Blanco, 2014; Bonilla et al., 2017; Donoso-Vázquez et al., 2017, 2018; Esteban & Gómez, 2022; Mena-Rodríguez & Velasco-Martínez, 2017; International Plan, 2020).

These studies also show that most common CVAWG behaviors committed among males is tied to myths of romantic love and the imposition of heteronormative canons of beauty (Blanco, 2014; Donoso-Vázquez et al., 2014, 2016, 2017). Meanwhile, despite committing fewer behaviors of cyberaggression in general, studies show that girls carry out CVAWG behaviors involving control (Donoso-Vázquez et al., 2018) and distancing themselves from the female sexual normativity (Esteban & Gómez, 2022; Villar et al., 2021).

For this part, the most common behaviors of cyberaggression related to CVAWG among boys and girls involve aspects of control, such as taking their partner’s mobile phone to review past activity, controlling their partner on social platforms, insulting a girl for being unattractive, and rating the physical appearance of women on websites or social networks (Blanco, 2014; Bonilla et al., 2017; Donoso-Vázquez et al., 2016, 2017; Esteban & Gómez, 2022; Vilà et al., 2018; Villar et al., 2021).

Research on cybervictimization reveals a worrisome pattern in the prevalence of CVAWG. Although most young people have not suffered these types of violence, among those who have, girls affirm to a greater degree having suffered this violence in all of the categories under review (Donoso-Vázquez et al., 2014, 2018; Vilà et al., 2015). Particularly, the most common behaviors of cyberaggression suffered by girls involve their partner controlling their mobile phone and their online activity, insulting them for being unattractive, or criticizing them for having multiple relationships (Blanco, 2014; Bonilla et al., 2017; Donoso-Vázquez et al., 2016, 2017, 2018; Vilà et al., 2015; Jiménez & Candela, 2021; Pineda, 2022; Villar et al., 2021). These practices reflect the persistence of a traditional relationship model, in which control is mistaken for affection under the influence of romantic love myths. At the same time, the pressure on girls to conform to sexist beauty standards affects their self-esteem and contributes to cyberaggression related to physical appearance, such as insults for being perceived as unattractive (Jiménez & Candela, 2021; Pineda, 2022).

In summary, we can conclude that he genders differences are particularly accentuated in the categories of myths of romantic love, the imposition of heteronormative canons of beauty, and other specific motivations. From a sociocognitive perspective, these differences can be explained by early differential socialization and the internalization of gendered sociocultural norms, which influence the likelihood of perpetrating or experiencing CVAWG (Ferrer & Bosch, 2019; Charkow & Nelson, 2000; Donoso-Vázquez et al., 2018; Vilà et al., 2018).

These gender differences align with institutional definitions of violence against women. A very important question is that, according to CEDAW General Recommendation No. 19 (CEDAW, 1992), violence against women and girls is a violence that is directed against a woman or girl because she is a woman or a girl (i.e., gender-based violence) or that affects women and girls disproportionately. In our case, and as the reviewed studies show, there are some types of cyber behaviors that are particularly suffered by girls, so we can consider it as CAWG.

Overall, the recognition that certain cyber behaviors disproportionately affect girls underscores the need to differentiate prevention strategies. This finding is particularly relevant in the Spanish context, where existing research on cyberaggression and cybervictimization reveal gender-specific patterns. Therefore, further exploration of the differences between boys and girls is essential in order to design more effective educational and preventive programs. Therefore, the analysis of these differences is the objective of the present study.

## 2. Materials and Methods

### 2.1. Participants

The study included the participation of 762 adolescents between 14 and 16 years of age, from nine public education centers in Mallorca. The sample included a greater number of girls than boys (n = 399 girls, n = 363 boys) representing 52.36% and 47.63%, respectively.

### 2.2. Procedure

This research falls within the framework of the interuniversity project “Gender violence 2.0” developed within the First Edition of the Call for Grants for Research Projects in Digital Humanities, sponsored by the BBVA Foundation and whose objective was deepened on the study of CVAWG in Spanish adolescents.

A quantitative methodology was used to carry out a diagnostic study in the form of an online survey, making it possible to gather specific data regarding the current situation of adolescents and their experience with CVAWG.

The random sample was obtained from clusters of students representing the educational centers in Mallorca. To ensure representativeness, the schools that were asked to participate were randomly selected. This selection was based on the official list of public schools. In each selected school, third- and fourth-year students of compulsory secondary education were asked to participate. All students in these levels who had attended class that day responded to the questionnaire.

The measuring instrument was administered electronically in the educational centers with an online response. In a time estimated of 45 min. This duration included the presentation of the research, the administration of the questionnaire (once related to cyberaggression committed, once related to the cybervictimization suffered), and a briefing, and varied depending on the pace of each group and the number of students present in the classroom.

At the beginning of the sessions, participants were informed that completing the questionnaire was entirely voluntary, that anonymity would be maintained, and that data confidentiality would be guaranteed. As a prerequisite, the school’s principal must sign an informed consent form.

### 2.3. Measures

The Gender Violence 2.0 Questionnaire (Vilà et al., 2015) was used, as it provides information on the cognitive and behavioral dimension of CVAWG among adolescents. Specifically, one of the five dimensions of the study gathers data on the CVAWG committed and suffered (Vilà et al., 2015) with 6 categories under study and 23 scale questions (Table 1). Participants indicate how often they have perpetrated this type cyberaggression and whether they have been cybervictims of them, using a 3-point Likert scale format: 1 (never), 2 (sometimes) and 3 (often), that could be technically considered as an ordinal scale of answer.

Although its authors do not provide any information on this, it can be stated that this questionnaire is reliable. Thus, for this sample, Cronbach’s alpha was calculated to estimate reliability, which was 0.859 when we used it to measure cyberaggression, and 0.884 when we used it to measure cybervictimization.

This study analyzes only 5 of the described categories. The results of the category for compulsory sexual heteronormativity (which includes items 5, 6, 7 and 11) were excluded from the results analysis as their singular focus does not fall within the scope of the objectives defined for this study because they are related to discrimination by sexual orientation or gender identity (not with CVAWG).

### 2.4. Design and Statistical Analysis

A descriptive analysis was performed for the number and percentage of responses obtained for each of the categories established for the boys and the girls. Based on the absolute or relative frequencies (percentages) obtained for each item, a ranking was prepared by category.

Following this step, contingency tables, the Chi-squared statistics, and the contingency coefficients were obtained. Crosstabs are a proper tool for analyzing the relationship between two or more categorical variables (that is, measured on a nominal or ordinal scale). In this sense, crosstabs were adequate for evaluating the association between a variable measured on a nominal scale (gender) and a variable measured on an ordinal scale (the frequency of cyberaggression and cybervictimization measured by the questionnaire used). When the Chi-squared test results were significant, the corrected typified residues (CTR) made it possible to identify in exactly which cases the association is undoubtedly significant.

The contingency coefficient is a measure of effect size and vary between 0 (indicating independence or absence of a relationship between the variables analyzed) and 1 (indicating a perfect association among the variables), whereby the greater the coefficient, the stronger the association between the variables. As indicated by Clark-Carter (2002), the contingency coefficient will never exceed a value of 1; even if the variables analyzed were completely dependent, their value will increase as the size of the table increases (in the case of a 2 × 2 table, the maximum value would be 0.707), while a value above 0.30 would indicate the existence of a good association between the variables.

It could be noted that we chose to analyze item by item for two reasons: firstly, because, although the items are thematically related, they are not factors; and secondly, because each item describes a form of CVAWG, so the analysis item by item is much more informative for the purpose of our research than obtaining a global score. Given this way of applying the questionnaire, its overall reliability has been indicated (not reliability by dimensions).

The gathered data were analyzed with the SPSS program (IBM Corp., Armonk, NY, USA), version 28.

## 3. Results

A notable result from the analysis of the frequency with which adolescents, boys and girls, have perpetrated CVAWG shows that the majority (greater than 70%) claimed not to have ever committed gender-based violence in virtual spaces (Table 2).

In general, the results from Table 2 indicate that when there is an association between gender and the CVAWG behavior analyzed, the boys claim to have perpetrated the behavior more often than girls, while the latter claim more often not to have ever perpetrated the behavior. Only in the case of Items 1, 18, 21 and 22 is there a reversal in the response pattern.

CVAWG behaviors related to sexual violence, such as obtaining compromising photos of a person to blackmail or take sexual advantage of them (Item 14), overloading somebody’s inbox with sexual content (Item 13) or sharing sexual videos/photos of a girl online without her permission (Item 15), are the types of cyberaggression least perpetrated by girls. These are followed by aggressive behavior driven by heteronormative canons of beauty, such as uploading a sexually objectified photo of a girl onto social platform (Item 10) or ridiculing a boy for having an unattractive appearance (Item 8). Likewise, criticizing somebody for their feminist ideology (Item 16) or sending images or making jokes about violence against women (Item 20) are not counted among the habitual behavior of girls.

On the other hand, the types of CVAWG behaviors least often perpetrated by boys, compared to girls, are those involving myths of romantic love, including forcing a partner to remove photos of friends on social platforms or to cease texting with somebody through WhatsApp (Item 22), taking a partner’s mobile to check phone calls and other activity (Item 21), and controlling a partner on social platforms (Item 18). Moreover, boys also report less participation in behaviors linked to deviation from the female sexual normativity, such as criticizing other girls for having multiple partners (Item 1).

There are several types of CVAWG behaviors that are quite often committed among adolescents, boys in particular, with an observable association between gender and the frequency with which they are committed (sometimes or often). According to the boys, they claim to a great extent to having engaged in cyber behaviors related to sexual violence and the imposing heteronormative canons of beauty. To a lesser extent, they also claim to have engaged in the behavior of an antipatriarchal and gender-stereotyped nature. The most common cyber behaviors related to sexual violence committed by boys include overloading somebody’s inbox with sexual content (Item 13), sharing sexual videos/photos of a girl online without her permission (Item 15), and obtaining compromising photos of a person to blackmail or take sexual advantage of them (Item 14). The most common behavior associated with the imposition of canons of beauty is criticizing a boy for not having a masculine appearance (Item 23). Regarding behaviors related to antipatriarchal manifestations and gender stereotypes, the most common is criticizing for their feminist ideology (Item 16) and sending images or making jokes about violence against women (Item 20).

In the case of girls, they claimed more frequently to have engaged in CVAWG related to myths of romantic love. Likewise, some admitted to engaging in controlling behavior on their partner’s social networks (Item 18), forcing their partner to remove photos of friends on Facebook or to stop texting with somebody through WhatsApp (Item 22). There was also an observable trend in criticizing other girls online for having multiple partners (Item 1), which reflects a deviation from the traditional female sexual normativity.

Given these results, there is an observable incidence of CVAWG behaviors from the perspective of the aggressor. Table 3 presents a ranking of cyber behavior least often committed by girls (in which the percentage of answers ‘never’ is significantly higher than boys) and for boys (in which the percentage of answers ‘never’ is significantly higher than girls). In contrast, Table 4 displays a ranking of cyber behaviors most often committed by girls (in which the percentage of answers ‘sometimes’ or ‘often’ is significantly higher than boys) and by boys (in which the percentage of answers ‘sometimes’ or ‘often’ is significantly higher than girls).

From the perspective of the victims, adolescents experience CVAWG at a very low rate. In fact, the majority (more than 70%) claim not to have been victim of CVAWG (Table 5).

In the case of association between gender and frequency of CVAWG, there is an observable general trend among boys claiming not to have been victims of the behavior in question, while the girls claim to have suffered the behavior with greater frequency. However, there is a reversal in the response pattern for Items 13 and 23.

Specifically, boys claim not to experience CVAWG related to myths of romantic love, the imposition of canons of beauty, deviation from the female sexual normativity, and sexual violence. Notable among control-based cyber behavior related to myths of romantic love, which boys claim not to have suffered, are forcing a partner to remove photos of friends on Facebook or to stop texting with somebody through WhatsApp (Item 22), allowing a partner to check phone activity (Item 21) or to control social platforms (Item 18). Among the types of cyberviolence related to canons of beauty, the behaviors least suffered include participating in websites that rate physical appearance (Item 9) and receiving insults for having an unattractive appearance (Item 8). Moreover, boys rarely experience CVAWG behaviors related to deviation from the female sexual normativity, such as criticism for having multiple partners (Item 1) or, conversely, for not having relationships (Item 3). Finally, the cyber behavior involving the use of threat against a girl to force her into a relationship (Item 12) is associated with sexual violence.

Conversely, girls experience greater victimization of CVAWG. They claim with greater frequency (“sometimes” or “often”) to have been controlled by their partner on social platforms (Item 18), to have been forced to remove photos with friends on Facebook or to stop texting with another person on WhatsApp (Item 22), and to have been insulted for having an unattractive appearance (Item 8). Notable among the CVAWG they claim to have suffered occasionally are cyberaggression involving deviation from the female sexual normativity, such as receiving criticisms or insults online for having multiple partners (Item 1) or, conversely, for not having relationships (Item 3). They also claim to have been victims of sexual violence, such as overloading their inbox with unsolicited sexual content (Item 13) or being threatened to enter into a relationship (Item 12). Similarly, they mention having been victim of symbolic violence by the imposition of canons of beauty on platforms where their bodies were rated (Item 9), and victim of CVAWG related to myths of romantic love, such as having their phones checked for activity (Item 21). Finally, adolescents claim to have been attacked in virtual environments for their feminist ideology (Item 16).

Additionally, the only CVAWG behaviors the boys claimed to have suffered to a greater extent than girls are the constant influx of emails containing sexual content (Item 13) (“often”) and to be criticized for not having a masculine appearance (Item 23) (“sometimes”).

## 4. Discussion

The results obtained indicate that, although the majority of adolescents to not engage in CVAWG, the boys record a greater prevalence than girls as cyberaggressors, which coincides with similar results noted in the literature on related topics (Bonilla et al., 2017; Donoso-Vázquez et al., 2014, 2016, 2017, 2018; Esteban & Gómez, 2022; Vilà et al., 2018; Villar et al., 2021). In line with other studies, the least perpetrated behaviors of cyberaggression are related to deviation from the female sexual normativity (such as criticizing a girl online for not being interested in boys) and sexual violence (such as blackmailing somebody with compromising photos to have sex and threatening a girl to engage in a romantic relationship) (Donoso-Vázquez et al., 2016, 2017; Villar et al., 2021).

Nevertheless, the low proportion of adolescents who admit to committing gender-based behaviors of cyberaggression demonstrates the presence of a discriminatory and hegemonic discourse of a patriarchal system in the cyber behavior of some adolescents (Rodríguez et al., 2020). VAWG is legitimized within a patriarchal system; thus, engaging in CVAWG behaviors reflects the acceptance of discriminatory social beliefs that sustain it. These behaviors indicate that cyberaggression among adolescents are rooted in patriarchal norms. Therefore, the low number of boys who admit to such acts confirms their existence. According to Ging (2019), the manosphere reinforces this discourse through digital environments that promote misogynistic and antifeminist ideas, normalize symbolic violence, and support sexist stereotypes and cyber control practices. This influence is especially concerning among boys, as these spaces offer narratives that justify or downplay violence against women, including CVAWG. This occurs during a especially sensitive life stage in which the identity and personal development of the aggressors and the victims (Donoso-Vázquez et al., 2017). From the perspective of developmental psychopathology, adolescence is a critical stage in which CVAWG may become a persistent relational pattern, affecting identity and socio-emotional development (Cicchetti & Rogosch, 2002). The internalization of gender roles adds complexity to the study of psychological disorders resulting from this form of digital violence from a gender-based perspective.

Advances in new technologies have exacerbated CVAWG, leading to new forms of cyberaggression, such as technology-facilitated partner violence. This has been identified as one of the most frequent types of violence against women, with a wide array of manifestations (Vázquez & Catalán, 2018). Moreover, in the period of time following the pandemic, the incidence rate increased by 76.4% among the adolescent population (November 2020–October 2021) (Fundación ANAR, 2023). The data demonstrate the persistence of a traditional relationship, reinforced by new and popular forms of technology-based control, such as monitoring a partner online and accessing their mobile phone (Blanco, 2014; Bonilla et al., 2017; Donoso-Vázquez et al., 2016, 2017, 2018; Vilà et al., 2015; Villar et al., 2021). Similarly, recent research has put forth other CVAWG behaviors, outside the scope of this study, but nevertheless reaffirming the diversity of technology-facilitated partner aggression and highlighting the need for their inclusion within the design of programs for the prevention of this violence. Examples include knowing the passwords for mobile phones and social platforms (Bonilla et al., 2017; Donoso-Vázquez et al., 2018; Vilà et al., 2015) and forcing a partner to remove photos from online platforms (Donoso-Vázquez et al., 2018; Vilà et al., 2015).

In this regard, it is surprising to note how insulting a girl for having an unattractive appearance has become the only cyberaggression related to the canons of beauty highlighted in this and other studies (Donoso-Vázquez et al., 2016, 2017, 2018; Esteban & Gómez, 2022; Villar et al., 2021), even when, according to scientific literature (Donoso-Vázquez et al., 2016, 2017; 2018, Esteban & Gómez, 2022; Vilà et al., 2015; Villar et al., 2021), it is also related to one of the categories with the largest variety of CVAWG behaviors, such as uploading humiliating photos or videos of overweight girls, posting photos of women as sexual objects (Donoso-Vázquez et al., 2018; Vilà et al., 2015) or rating the physical appearance of a woman online (Donoso-Vázquez et al., 2016, 2017; Esteban & Gómez, 2022; Villar et al., 2021), the latter being the most popular act of aggression. In this regard, preventative programs should incorporate socially critical strategies (Lardies & Potes, 2022) that can lead to a reduction in the most universal forms of CVAWG (Pineda, 2022), which particularly affects girls, who face greater pressure to adjust to the stereotypes of feminine beauty. They must learn to identify the messages and images that show (or modify) bodies according to social desirability, as well as those that promote the sexualization of women and relate beauty with the success of women (Jiménez & Candela, 2021). We must be attentive to digital content that mainstreams and reaffirms references of beauty canons, influencing the perceptions and expectations of boys and girls, and discriminating against those who fall short of the idealized normative, even when the majority of cases are in fact unrealities filtered through social platforms. For this reason, countries such as Norway, France and the United Kingdom have already established legislation prohibiting the unauthorized publication of retouched or filtered photographs (Mas, 2021), therefore reducing the feelings of frustration from seeing unrealistic ideals of beauty (Ruiz, 2022), which are unattainable without the use of digital enhancement tools. These actions must be accompanied by programs that foster sympathy and tolerance for the esthetic plurality of women and their physical appearance, which could influence the mindset of an aggressor and help him reconsider the motivation behind his cyberattacks.

Among the CVAWG frequently committed by boys are those related to sexual violence, the imposition of canons of beauty, deviation from the feminine normativity, and stereotyped antipatriarchal manifestations. Similar studies coincide that the most common actions of cyberaggression committed by boys are those driven by sexual violence (Ferreiro et al., 2016; Villar et al., 2021) and for having a feminist ideology (Donoso-Vázquez et al., 2018; Esteban & Gómez, 2022; Villar et al., 2021).

It is worth noting that, according to current figures, some forms of CVAWG such as cybercrimes against sexual freedom are on the rise, having increased by 10.7% in 2022 from the previous year, especially among men, with extremely high rates of sexual cyberdelinquency. Even among minors in the 14–17 age group, 96% of all arrests were related to sexual cyberdelinquency (Ministry of Interior, 2023). This indicates a strong need to establish programs for the prevention of sexual violence primarily targeting adolescent boys, paying special attention to the most frequently committed acts of cyberaggression such as overloading an inbox with sexual content, the online dissemination of unauthorized videos/photographs of a sexual nature, and using compromising photographs to blackmail and take sexual advantage of a girl (Ferreiro et al., 2016; Villar et al., 2021). Beyond representing a serious violation of victims’ privacy and dignity, these behaviors have substantial implications for the health of adolescent girls, who are particularly vulnerable to this form of violence. As reported by Longobardi et al. (2021), experiences of online sexual victimization are linked to elevated levels of psychological distress, anxiety, depression, and social isolation, along with diminished self-esteem and a deterioration in interpersonal relationships. Collectively, these outcomes may adversely affect adolescents’ psychosocial development and academic achievement, thereby reinforcing the urgent need for gender-sensitive educational interventions focused on both prevention and harm mitigation.

Notable among CVAWG related to the imposition of canons of beauty is the concept of an ideal masculine beauty, weighing heavily among boys and serving as the primary motivating force of their criticism against other boys who deviate from these esthetic canons and lack a strong masculine appearance. Among cyber behaviors related to antipatriarchal manifestations, the boys tend to attack feminist ideology more frequently and ridicule sexual assaults against women. This trend has been noted in various studies (Delgado Ontivero & Sánchez-Sicilia, 2023; Ferreiro et al., 2016; Villar Aguilés & Pecourt, 2021) indicating a continually increasing antifeminist stand among adolescents in virtual spaces due to, among other factors, the influence of misogynist and antifeminist discourse present in the manosphere on young boys from an early age (Borraz, 2022; García-Mingo et al., 2022). It is necessary to work with boys to dismantle the false narratives employed by the manosphere subculture to propagate antifeminist attitudes and attacks, and exploit women as sexual objects (García-Mingo & Díaz, 2022). An urgent challenge for prevention, as the manosphere not only reproduces problematic content but also shapes masculine identities that feel threatened by progress toward gender equality (Ging, 2019).

In the case of girls, the results obtained coincide with other studies indicating the most frequently committed cyber behaviors to be related to myths of romantic love (Donoso-Vázquez et al., 2018; Villar et al., 2021) and with the deviation from the female sexual normativity (Esteban & Gómez, 2022; Villar et al., 2021). Most notable among the latter category is to criticize a girl online for having multiple partners, which demonstrates the influence of certain romantic myths on the understanding of romantic partners. Internalizing myths on romantic partners, exclusivity and fidelity may lead some to assume that in love, the passionate, romantic and erotic submission should be exclusive to one person, generally one’s partner. This model of a monogamous relationship reinforces an ideal of a dating relationship that condemns any deviation from a preestablished social norm (Bosch et al., 2013; Ferreiro et al., 2016), which may discredit those who do not conform to this pattern, especially with regard to women.

According to this study, the most common control-based CVAWG behaviors among girls include controlling their partner on social platforms, checking their partner’s phone activity, and forcing their partner to remove photos of friends on Facebook or to stop sending text messages through WhatsApp. However, the differences observed among the studies cannot be disregarded. Some results suggest that girls commit more cyber-controlling acts of aggression, while other data indicate, conversely, that these types of behavior are primarily carried out by boys (Rebollo-Catalán et al., 2018). Moreover, while some studies note no significant differences between girls and boys, others highlight the most common types of control-based cyber behavior for each gender (Bonilla et al., 2017).

Regarding the results that indicate girls are more controlling online behavior various authors agree that, instead of eliminating controlling behavior of boys, girls are in fact adopting the same behavior (Barlett & Coyne, 2014; Donoso-Vázquez et al., 2018; García et al., 2016), based on the idea that “girls are being held hostage by the patriarchy towards violent behaviors previously committed by the masculine collective” (Donoso-Vázquez et al., 2017, p. 15). However, this claim requires a more in-depth gender analysis to avoid any interpretations suggestive of a supposed bidirectionality of gender violence, a characteristic discourse held by the far right and the assertion of neosexism (Donoso-Vázquez & Prado, 2014). The study of the disparities of patriarchal power and dominance in dating relationships (Sanmartín-Andújar et al., 2023) shows how the consequences of control have a more negative effect on women (Borrajo et al., 2015; Estébanez & Vázquez, 2013; Torres et al., 2013). This is partly due to a greater psychological vulnerability among women within the relationship and social fabric (Donoso-Vázquez et al., 2014; Donoso-Vázquez et al., 2017), the influences of romantic myths, and love as a gender identity (Lagarde, 2001). In this regard, coping strategies are developed from an early age according to the lessons learned for love relationships, which for girls is the main objective and focal point of their identity (Lagarde, 2001). According to myths of romantic love, girls internalize a sense of duty, patience, obligation and endurance for the sake of love, such as what occurs with controlling behavior (Ferrer & Bosch, 2013, 2019), while boys learn strategies enabling them to avoid these behaviors. Proof of this is that girls tend to justify control as a sign of affection or interest, while boys are less tolerant to being controlled and, consequently, reject these acts more firmly (Sortzen Consultoría, 2010).

Another interpretation of these results would involve exploring the causes leading girls and boys to engage in CVAWG and whether, in the case of girls, these cyber behaviors are in direct response to the same control they have received (Donoso-Vázquez et al., 2017). Nevertheless, the key issue is understanding why boys rarely admit to committing, being victim to, or witnessing CVAWG in their relationship, especially in the case of cybervictimization (Donoso-Vázquez et al., 2018; Ferreiro, 2024). It is surprising that while girls claim to exert greater control over their partners, boys do not consider themselves victims of control-based cyber behavior, an issue that will also be discussed in greater detail later. This phenomenon can be partly explained by trait online disinhibition, understood as the tendency to act more impulsively or aggressively in digital environments due to perceived anonymity and lack of consequences (Kasturiratna & Hartanto, 2024). From a gender perspective, this trait tends to reinforce patriarchal dynamics. In the case of boys, socialization based on dominance may promote cyberaggressive behaviors, while for girls, influenced by romantic love myths, it may lead to controlling behaviors as a form of response.

Regarding cybervictimization, the results obtained indicate that the majority of adolescents claim not to have suffered CVAWG. Nevertheless, among those who have, girls are more often the victim of cyberaggression than boys, as highlighted in various studies (Donoso-Vázquez et al., 2014, 2018; Vilà et al., 2018).

As noted in this study, the CVAWG most commonly suffered by girls is driven by myths of romantic love, the female sexual normativity, and sexual violence (Blanco, 2014; Bonilla et al., 2017; Donoso-Vázquez et al., 2018; Donoso-Vázquez et al., 2017; Esteban & Gómez, 2022; Mena-Rodríguez & Velasco-Martínez, 2017; Vilà et al., 2015). The behavior most commonly suffered by boys included criticism for not having a very masculine appearance and overloading their inbox with sexual context. Both types of behavior should be addressed in greater detail among boys, as they are most often the perpetrators of these acts of violence, as we have already seen.

Forms of control-based CVAWG behaviors most commonly suffered by adolescents involve controlling their partner’s social platforms, forcing them to remove photographs of friends or to stop texting via WhatsApp, and checking their phone activity. A wide array of acts identified in this and other studies reinforcing the concept of romantic love as one of the primary motivations behind the cyberaggression suffered by adolescents (Blanco, 2014; Bonilla et al., 2017; Donoso-Vázquez et al., 2018; Donoso-Vázquez et al., 2017; Esteban & Gómez, 2022; Mena-Rodríguez & Velasco-Martínez, 2017). These studies highlight the high capacity of adolescents to identify control-based CVAWG behaviors, which, when added to their ability to identify this behavior as acts of violence (Ferreiro, 2024), seem to suggest that they are able to recognize a toxic relationship. That said, it would be convenient to study whether girls are sufficiently equipped with the best tools to sever violent dating relationships.

Surprisingly, while the girls claim to engage in more control-based CVAWG than the boys, they do not see themselves as victims of these cyber behaviors. On the other hand, while girls experience cyberviolence driven by myths of romantic love, the boys do not see themselves as responsible for these types of cyberviolence (Blanco, 2014; Bonilla et al., 2017; Donoso-Vázquez et al., 2017, 2018; Esteban & Gómez, 2022; Mena-Rodríguez & Velasco-Martínez, 2017). It would be relevant to analyze whether girls identify control-based cyber behaviors, as victims and aggressors, due to a greater perception of violence in romantic love than boys, who normalize their controlling role in their relationship within “a patriarchal society where the boys exert violence and the girls suffer” (Bonilla et al., 2017, p. 60). From a sociocognitive perspective, these differences stem from differential gender socialization, which promotes the early internalization of patriarchal norms (Ferrer & Bosch, 2019; Charkow & Nelson, 2000; Donoso-Vázquez et al., 2018; Vilà et al., 2018). Girls are taught to be compliant, while boys assimilate messages of dominance and possession within affective relationships (Bosch et al., 2013). The results also support the pyramid model (Ferrer & Bosch, 2019), which argues that behavioral differences between men and women are not solely the result of biological factors, but are socially constructed through discourse, cultural practices, and patriarchal power relations, which are also present in digital contexts. In this sense, naturalization differentiated from domination and control-driven relationships guarantee a social order (Bourdieu, 2000) in which men minimize the violence committed and maximize the suffering, while women do the opposite, minimizing the violence suffered and maximizing the violence committed (Kimmel, 2006). Another possibility could be the influence of social desirability, prompting boys to deny or conceal these acts, while admitting to other acts of cyberaggression such as sexual violence. In either case, it is critical to develop programs that deconstruct false beliefs of love, control and violence.

Several control-based cyber behaviors have been associated with female sexual normativity, such as being criticized on social platforms for having multiple partners or for not engaging in intimate behavior with boys. Likewise, online insults directed at girls for their unattractive physical appearance constitute one of the most frequent forms of cyberaggression. Previous studies (Donoso-Vázquez et al., 2017; Esteban & Gómez, 2022; Mena-Rodríguez & Velasco-Martínez, 2017) have obtained similar results, although they have not separated the information according to gender, which makes it difficult to determine exactly whether this is also one of the most prevalent acts among girls. Even so, they are among the most recurrent in digital environments.

Surprisingly, previous studies employing data-gathering tools similar to those used in this study do not highlight technology-facilitated sexual violence as one of the most frequent types of cyberviolence in which girls see themselves as victims. However, this study notes that a recurrence of sexual content received through email and threats to engage in a relationship are relatively common experiences, in line with other evidence. (International Plan, 2020). Also of significance is that adolescent girls do not identify as victims of sexual violence, despite data indicating that 9 of every 10 victims of sexual cyberviolence in 2023 were women and that, in the case of minors, this figure is nearly four times the cases among boys (CIS, 2023). In turn, boys claim to have committed these acts far more frequently.

Finally, girls are also victims of cyberviolence linked to canons of beauty, such as being rated on appearance rating websites, supporting a feminist ideology, and other aggressive acts perpetrated primarily by boys, according to this and other studies (Donoso-Vázquez et al., 2018; Esteban & Gómez, 2022; Ferreiro et al., 2016; Villar et al., 2021).

This study presents several limitations that should be addressed in future research. First, given the rapid advancement of technology, it is essential to continuously review the evolution of violent cyber behaviors in order to maintain the relevance and validity of both the data and the methodological approaches used. In this regard, it will also be necessary to pay attention to emerging risks associated with the use of artificial intelligence (AI), particularly concerning the production, dissemination, and automation of content that may reproduce or amplify CVAWG. Secondly, the study relied exclusively on a quantitative methodology based on self-reports, which entails risks related to social desirability bias, subjectivity, and memory inaccuracies. It would be advisable to complement this with qualitative methods to delve more deeply into certain aspects, such as the reasons why boys claim not to commit or experience CVAWG linked to the myths of romantic love. Another significant limitation is the lack of standardized terminology to adequately categorize the various forms of cyberviolence, especially in the ability to distinguish between gender-based partner violence and other CVAWG behaviors. A standardized terminology will simplify research and academic collaboration. In turn, although the present study covers a vast and diverse sample of adolescents, the analysis focused on a general comparison between boys and girls, which would allow future research to examine the impact of sociodemographic variables such as age, socio-economic level or family structure. It would also be interesting to expand this study to other countries and examine whether the findings can be generalized. Finally, although the questionnaire employed in this study is a pioneering and thoroughly validated tool, some results suggest that it must be updated to adapt to the evolution of CVAWG, and to include some other forms of cyberviolence such as sexting, grooming, etc.

In short, in the current context, where Information and Communication Technologies (ICT) have transformed the social interaction of the native digital generations, there is a worrying trend towards the normalization of CVAWG behaviors content and uses of social networks from a very early age. Therefore, it is essential to continue investigating these dynamics from an intersectional perspective that considers both gender differences and the sociocultural conditioning factors that perpetuate these types of violence. Only then will it be possible to design effective preventive strategies that contribute to eradicating these acts of cyberaggression and promote more egalitarian online behavior. Likewise, it will be crucial to develop preventive educational strategies contributing to the safe and responsible use of social networks, especially among young people.

From a practical standpoint, the findings of this study underscore the importance of implementing targeted, gender-specific educational interventions that address CVAWG from early adolescence. In the case of boys, particular emphasis should be placed on preventing the most frequently reported forms of cyberaggression, such as those associated with sexual violence, controlling behaviors, and antifeminist discourse. For girls, it is essential to promote awareness of cybervictimization linked to romantic relationships, internalized beauty standards, and myths of romantic love, by fostering recognition, empowerment, and the capacity to disengage from harmful relational dynamics. These interventions should adopt a gender-responsive approach that acknowledges the structural inequalities contributing to girls’ increased vulnerability, while avoiding simplistic or misleading interpretations of violence as symmetrical or bidirectional. Consequently, coordinated efforts among educational, community, and healthcare institutions are recommended, including the provision of specialized training for teaching staff, the establishment of early detection protocols, and the development of integrated support systems. A multidisciplinary strategy adapted to the complexities of the current digital context is essential to mitigate the impact of CVAWG during adolescence and to promote healthier forms of digital interaction.

## Figures and Tables

**Table 1:** Categories of dimension for CVAWG experiences in the Gender Violence 2.0 Questionnaire (adapted from [Donoso-Vázquez et al., 2017]).

Dimension	Categories
Experiences of sexist violence in virtual environments or CVAWG	Deviation from the female sexual normativity. Transgression of compulsory sexual heteronormativity. Imposition of heteronormative canons of beauty. Sexual violence. Violence related to myths of romantic love. Other.

**Table 2:** Frequency with which adolescents have perpetrated CVAWG.

	ITEMS	Girls (n = 399) Boys (n = 363)	Never	Sometimes	Often	Significance
Deviation from the female sexual normativity	(Item 1) Use the internet to criticize a girl for having multiple partners.	Boys n (%) CTR	311 (85.7%) 2.9	48 (13.2%) −2.4	4 (1.1%) −1.6	Chi2(2) = 9.160 *p* = 0.010 CC = 0.109
		**Girls** n (%) CTR	309 (77.4%) −2.9	79 (19.8%) 2.4	11 (2.8%) 1.6	
		Total n (%)	680 (81.4%)	127 (16.7%)	15 (25%)	
	(Item 2) Use a mobile or social network platforms to bully a girl for being provocative.	Boys n (%) CTR	336 (92.6%) 0.6	23 (6.3%) −0.9	4 (1.1%) 0.9	Chi2(2) = 1.642 *p* = 0.220 CC = 0.046
		**Girls** n (%) CTR	365 (91.5%) −0.6	32 (8%) 0.9	2 (0.5%) −0.9	
		Total n (%)	701 (92%)	55 (7.2%)	6 (0.8%)	
	(Item 3) Use the internet to criticize a girl for not being interested in boys.	Boys n (%) CTR	353 (97.2%) −1.2	8 (2.2%) 1	2 (0.6%) 0.7	Chi2(2) = 1.473 *p* = 0.479 CC = 0.044
		**Girls** n (%) CTR	393 (98.5%) 1.2	5 (1.3%) −1	1 (0.3%) −0.7	
		Total n (%)	746 (97.9%)	13 (1.7%)	3 (0.4%)	
	(Item 4) Use the internet to insult a girl for not being intimate with boys.	Boys n (%) CTR	358 (98.6%) −0.5	4 (1.1%) 0.5	1 (0.3%) 0.1	Chi2(2) = 0.261 *p* = 0.878 CC = 0.018
		Girls n (%) CTR	395 (99%) 0.5	3 (0.8%) −0.5	1 (0.3) −0.1	
		Total n (%)	753% (98.8%)	7 (0.9%)	2 (0.3%)	
Imposition of heteronormative canons of beauty	(Item 8) Insult a girl for having an unattractive appearance.	Boys n (%) CTR	303 (83.5%) −1.1	55 (15.2%) 0.9	5 (1.4%) 0.5	Chi2(2) = 1.162 *p* = 0.559 CC = 0.039
		**Girls** n (%) CTR	344 (86.2%) 1.1	51 (12.8%) −0.9	4 (1%) −0.5	
		Total n (%)	647 (84.9%)	106 (13.9%)	9 (1.2%)	
	(Item 9) Create, participate in or view an appearance rating website to judge a girl’s physical appearance.	Boys n (%) RTC	319 (87.9%) 0.2	41 (11.3%) 0.2	3 (0.8%) −1.1	Chi2(2) = 1.297 *p* = 0.523 CC = 0.041
		**Girls** n (%) CTR	349 (87.5%) −0.2	43 (10.8%) −0.2	7 (1.8%) 1.1	
		Total n (%)	668 (87.7%)	84 (11%)	10 (1.3%)	
	(Item 10) Upload a sexually objectified photo of a girl to Facebook or other social network platforms.	Boys n (%) CTR	332 (91.5%) −4.3	29 (8%) 4.1	2 (0.6%) 1.5	Chi2(2) = 18.758 *p* < 0.010 CC = 0.155
		Girls n (%) CTR	392 (98.2%) 4.3	7 (1.8%) −4.1	0 (0.0%) −1.5	
		Total n (%)	724 (95%)	36 (4.7%)	2 (0.3%)	
	(Item 23) Criticize a boy for not having a masculine appearance.	Boys n (%) RTC	295 (81.3%) −2.9	59 (16.3%) 2.2	9 (2.5%) 2.3	Chi2(2) = 10.651 *p* = 0.005 CC = 0.117
		**Girls** n (%) CTR	354 (88.7%) 2.9	43 (10.8%) −2.2	2 (0.5%) −2.3	
		Total n (%)	649 (85.2%)	102 (13.4%)	11 (1.4%)	
Sexual violence	(Item 12) Threaten a girl to enter into a relationship.	Boys n (%) CTR	357 (98.3%) 0.1	5 (1.4%) −0.1	1 (0.3%) 0.1	Chi2(2) = 0.026 *p* = 0.987 CC = 0.006
		**Girls** n (%) CTR	392 (98.2%) −0.1	6 (1.5%) 0.1	1 (0.3%) −0.1	
		Total n (%)	749% (98.3%)	11 (1.4%)	2 (0.3%)	
	(Item 13) Overload somebody’s inbox with sexual content.	Boys n (%) CTR	344 (94.8%) −4.0	14 (3.9%) 3.2	5 (1.4%) 2.4	Chi2(2) = 16.126 *p* < 0.001 CC = 0.144
		**Girls** n (%) CTR	397 (99.5%) 4	2 (0.5%) −3.2	0 (0.0%) −2.4	
		Total n (%)	741 (97.2%)	16 (2.1%)	5 (0.7%)	
	(Item 14) Obtain compromising photos of a person to blackmail or take sexual advantage of them.	Boys n (%) CTR	356 (98.1%) −2.8	4 (1.1%) 2.1	3 (0.8%) 1.8	Chi2(2) = 7.766 *p* = 0.021 CC = 0.100
		**Girls** n (%) CTR	399 (100%) 2.8	0 (0.0%) −2.1	0 (0.0%) −1.8	
		Total n (%)	755 (99.1%)	4 (0.5%)	3 (0.4%)	
	(Item 15) Share sexual videos/photos of a girl online without her permission.	Boys n (%) CTR	341 (93.9%) −3.3	20 (5.5%) 3.6	2 (0.6%) 0.1	Chi2(2) = 12.678 *p* = 0.002 CC = 0.128
		**Girls** n (%) CTR	393 (98.5%) 3.3	4 (1%) −3.6	2 (0.5%) −0.1	
		Total n (%)	734 (96.3%)	24 (3.1%)	4 (0.5%)	
Violence associated with romantic love	(Item 18) Control your partner on Facebook, Twitter…	Boys n (%) CTR	311 (85.7%) 5.1	46 (12.7%) −4	6 (1.7%) −3.1	Chi2(2) = 27.816 *p* < 0.001 CC = 0.188
		**Girls** n (%) CTR	280 (70.2%) −5.1	95 (23.8%) 4	24 (6%) 3.1	
		Total n (%)	591 (77.6%)	141 (18.5%)	30 (3.9%)	
	(Item 19) Use your partner’s password to block friends on social network platforms.	Boys n (%) CTR	347 (95.6%) 1.5	15 (4.1%) −1.2	1 (0.3%) −1.2	Chi2(2) = 2.985 *p* = 0.225 CC = 0.062
		**Girls** n (%) CTR	371 (93%) −1.5	24 (6%) 1.2	4 (1%) 1.2	
		Total n (%)	718 (94.2%)	39 (5.1%)	5 (0.7%)	
	(Item 21) Take your partner’s mobile to check phone calls and other activity.	Boys n (%) CTR	327 (90.1%) 4.5	32 (8.8%) −4.3	4 (1.1%) −1	Chi2(2) = 19.930 *p* < 0.001 CC = 0.160
		**Girls** n (%) CTR	312 (78.2%) −4.5	79 (19.8%) 4.3	8 (2%) 1	
		Total n (%)	639 (83.9%)	111 (14.6%)	12 (1.6%)	
	(Item 22) Force your partner to remove photos of friends on Facebook, or to stop texting with somebody through WhatsApp.	Boys n (%) CTR	341 (93.9%) 2.5	20 (5.5%) −2.1	2 (0.6%) −1.5	Chi2(2) = 6.922 *p* = 0.031 CC = 0.095
		**Girls** n (%) CTR	354 (88.7%) −2.5	38 (9.5%) 2.1	7 (1.8%) 1.5	
		Total n (%)	695 (91.2%)	58 (7.6%)	9 (1.2%)	
Other (Gender stereotypes and expression of antipatriarchal position)	(Item 16) Use internet to criticize somebody for their feminist ideology.	Boys n (%) CTR	326 (89.8%) −3.9	33 (9.1%) 3.6	4 (1.1%) 1.5	Chi2(2) = 14.989 *p* < 0.001 CC = 0.139
		**Girls** n (%) CTR	386 (96.7%) 3.9	12 (3%) −3.6	1 (0.3%) −1.5	
		Total n (%)	712 (93.4%)	45 (5.9%)	5 (0.7%)	
	(Item 17) Kick somebody out of a chat or forum for being female.	Boys n (%) CTR	355 (97.8%) −1.7	5 (1.4%) 0.8	3 (0.8%) 1.8	Chi2(2) = 4.047 *p* = 0.132 CC = 0.073
		**Girls** n (%) CTR	396 (99.2%) 1.7	3 (0.8%) −0.8	0 (0.0%) −1.8	
		Total n (%)	751 (98.6%)	8 (1%)	3 (0.4%)	
	(Item 20) Send images or make jokes about violence against women.	Boys n (%) CTR	316 (87.1%) −5.8	40 (11%) 5.5	7 (1.9%) 1.8	Chi2(2) = 34.240 *p* < 0.001 CC = 0.207
		**Girls** n (%) CTR	391 (98%) 5.8	6 (1.5%) −5.5	2 (0.5%) −1.8	
		Total n (%)	707 (92.8%)	46 (6%)	9 (1.2%)	

CTR: corrected typified residues.

**Table 3:** Ranking cyber behaviors is least often committed by boys and girls.

	BOYS	GIRLS
1	Force your partner to remove photos of friends on Facebook, or to stop texting with somebody through WhatsApp. (Item 22).	Obtain compromising photos of a person to blackmail or take sexual advantage of them. (Item 14).
2	Take your partner’s mobile to check phone calls and other activities. (Item 21).	Overloading somebody’s inbox with sexual content. (Item 13).
3	Control your partner on social networks. (Item 18). Use the internet to criticize a girl for having multiple partners (Item 1).	Share sexual videos/photos of a girl online without her permission. (Item 15).
4		Upload a sexually objectified photo of a girl to Facebook or other social network platforms. (Item 10).
5		Send messages or make jokes about violence against women (Item 20).
6		Use the internet to criticize somebody for their feminist ideology. (Item 16).
7		Criticize a boy for not having a masculine appearance. (Item 23).

Note: If two or more items are listed in the same position is because there is a tie among them.

**Table 4:** Ranking cyber behavior is most often committed by girls and boys.

	BOYS	GIRLS
1	Criticize a boy for not having a masculine appearance (sometimes and often) (Item 23).	Control your partner on social networks (sometimes and often) (Item 18).
2	Fill somebody’s mailbox with sexual content (sometimes and often) (Item 13).	Take your partner’s mobile to check phone calls and other activities (sometimes) (Item 21). Use the internet to criticize a girl for having multiple partners (sometimes) (Item 1).
3	Send messages or joke about violence against women (sometimes) (Item 20).	Force your partner to remove photos of friends on Facebook, or to stop texting with somebody through WhatsApp (sometimes) (Item 22).
4	Use the internet to criticize somebody for their feminist ideology (sometimes) (Item 16).	
5	Upload a sexually objectified photo of a girl to Facebook or other social network platforms. (Item 10).	
6	Share sexual videos/photos of a girl online without her permission (sometimes) (Item 15).	
7	Obtain compromising photos of a person to blackmail or take sexual advantage of them (sometimes) (Item 14).	

Note: If two or more items are listed in the same position is because there is a tie among them.

**Table 5:** Frequency with which male and female adolescents have suffered CVAWG.

	ITEMS	Girls (n = 399) Boys (n = 363)	Never	Sometimes	Often	Significance
Deviation from the female sexual normativity	(Item 1) Use the internet to criticize a girl for having multiple partners.	Boys n (%) CTR	332 (91.5%) 2.6	26 (7.2%) −2.3	5 (1.4%) −1.1	Chi2(2) = 6.642 *p* = 0.036 CC = 0.093
		Girls n (%) CTR	341 (85.5%) −2.6	48 (12%) 2.3	10 (2.5%) 1.1	
	(Item 2) Use a mobile or social network platforms to bully a girl for being provocative.	Boys n (%) CTR	338 (93.1%) −1.8	20 (5.5%) 1.3	5 (1.4%) 1.3	Chi2(2) = 3.460 *p* = 0.177 CC = 0.067
		Girls n (%) CTR	383 (96%) 1.8	14 (3.5%) −1.3	2 (0.5%) −1.3	
	(Item 3) Use the internet to criticize a girl for not being interested in boys.	Boys n (%) CTR	347 (95.6%) 1.7	12 (3.3%) −2.2	4 (1.1%) 0.9	Chi2(2) = 5.485 *p* = 0.064 CC = 0.085
		Chicas n (%) CTR	370 (92.7%) −1.7	27 (6.8%) 2.2	2 (0.5%) −0.9	
	(Item 4) Use the internet to insult a girl for not being intimate with boys.	Boys n (%) CTR	350 (96.4%) 3.6	11 (3%) −3.3	2 (0.6%) −1.3	Chi2(2) = 12.940 *p* = 0.002 CC = 0.129
		Girls n (%) CTR	358 (89.7%) −3.6	35 (8.8%) 3.3	6 (1.5%) 1.3	
Imposition of heteronormative canons of beauty	(Item 8) Insult a girl for having an unattractive appearance.	Boys n (%) CTR	332 (91.5%) 6.7	25 (6.9%) −5.7	6 (1.7%) −3.1	Chi2(2) = 44.762 *p* < 0.001 CC = 0.236
		Girls n (%) CTR	290 (72.7%) −6.7	85 (21.3%) 5.7	24 (6%) 3.1	
	(Item 9) Create, participate in or view an appearance rating website to judge a girl’s physical appearance.	Boys n (%) CTR	339 (93.4%) 2.7	19 (5.2%) −3	5 (1.4%) 0.5	Chi2(2) = 9.169 *p* = 0.010 CC = 0.109
		Girls n (%) CTR	350 (87.7%) −2.7	45 (11.3%) 3	4 (1%) −0.5	
	(Item 10) Upload a sexually objectified photo of a girl to Facebook or other social network platforms.	Boys n (%) CTR	345 (95%) −0.6	14 (3.9%) 0.1	4 (1.1%) 1.5	Chi2(2) = 2.122 *p* = 0.346 CC = 0.053
		Girls n (%) CTR	383 (96%) 0.6	15 (3.8%) −0.1	1 (0.3%) −1.5	
	(Item 23) Criticize a boy for not having a masculine appearance.	Boys n (%) CTR	327 (90.1%) −2.9	28 (7.7%) 2.4	8 (2.2%) 1.7	Chi2(2) = 8.640 *p* = 0.013 CC = 0.106
		Girls n (%) CTR	381 (95.5%) 2.9	15 (3.8%) −2.4	3 (0.8%) −1.7	
Direct and indirect sexual violence	(Item 12) Threaten a girl to enter into a relationship.	Boys n (%) CTR	354 (97.5%) 2.8	6 (1.7%) −3.1	3 (0.8%) 0.1	Chi2(2) = 9.567 *p* = 0.008 CC = 0.111
		Girls n (%) CTR	372 (93.2) −2.8	24 (6%) 3.1	3 (0.8%) −0.1	
	(Item 13) Overloading somebody’s inbox with sexual content.	Boys n (%) CTR	338 (93.1%) −0.1	17 (4.7%) −1.1	8 (2.2%) 2.5	Chi2(2) = 7.272 *p* = 0.026 CC = 0.097
		Girls n (%) CTR	372 (93.2%) 0.1	26 (6.5%) 1.1	1 (0.3%) −2.5	
	(Item 14) Obtain compromising photos of a person to blackmail or take sexual advantage of them.	Boys n (%) CTR	354 (97.5%) 0.4	5 (1.4%) −1.1	4 (1.1%) 0.9	Chi2(2) = 2.107 *p* = 0.349 CC = 0.053
		Girls n (%) CTR	387 (97%) −0.4	10 (2.5%) 1.1	2 (0.5%) −0.9	
	(Item 15) Share sexual videos/photos of a girl online without her permission.	Boys n (%) CTR	347 (95.6%) −1.2	14 (3.9%) 0.9	2 (0.6%) 1.5	Chi2(2) = 2.953 *p* = 0.228 CC = 0.062
		Girls n (%) CTR	388 (97.2%) 1.2	11 (2.8%) −0.9	0 (0.0%) −1.5	
Violence associated with myths of romantic love	(Item 18) Control your partner on Facebook, Twitter…	Boys n (%) CTR	321 (88.4%) 4.9	36 (9.9%) −4	6 (1.7%) −2.7	Chi2(2) = 24.928 *p* < 0.001 CC = 0.178
		Girls n (%) CTR	297 (74.4%) −4.9	81 (20.3%) 4	21 (5.3%) 2.7	
	(Item 19) Use your partner’s password to block friends on social network platforms.	Boys n (%) CTR	336 (92.6%) 0.8	20 (5.5%) −0.3	7 (1.9%) −1	Chi2(2) = 1.024 *p* = 0.599 CC = 0.037
		Girls n (%) CTR	363 (91%) −0.8	24 (6%) 0.3	12 (3%) 1	
	(Item 21) Take your partner’s mobile to check phone calls and other activity.	Boys n (%) CTR	325 (89.5%) 4.1	31 (8.5%) −3.6	7 (1.9%) −1.7	Chi2(2) = 16.487 *p* < 0.001 CC = 0.146
		Girls n (%) CTR	314 (78.8%) −4.1	69 (17.3%) 3.6	16 (4%) 1.7	
	(Item 22) Force your partner to remove photos of friends on Facebook, or to stop texting with somebody through WhatsApp.	Boys n (%) CTR	340 (93.7%) 3.7	17 (4.7%) −2.9	6 (1.7%) −2.1	Chi2(2) = 13.523 *p* = 0.001 CC = 0.132
		Girls n (%) CTR	341 (85.5%) −3.7	41 (10.3%) 2.9	17 (4.3%) 2.1	
Other (Gender stereotypes and expression of antipatriarchal position)	(Item 16) Use internet to criticize somebody for their feminist ideology.	Boys n (%) CTR	347 (95.6%) 4.1	11 (3%) −4.3	5 (1.4%) −0.4	Chi2(2) = 18.476 *p* < 0.001 CC = 0.154
		Girls n (%) CTR	348 (87.2%) −4.1	44 (11%) 4.3	7 (1.8%) 0.4	
	(Item 17) Kick somebody out of a chat or forum for being female.	Boys n (%) CTR	352 (97%) 1.2	9 (2.5%) −1.7	2 (0.6%) 1.5	Chi2(2) = 4.953 *p* = 0.084 CC = 0.080
		Girls n (%) CTR	380 (95.2%) −1.2	19 (4.8%) 1.7	0 (0.0%) −1.5	
	(Item 20) Send images or make jokes about violence against women.	Boys n (%) CTR	331 (91.2%) 0.2	24 (6.6%) −0.7	8 (2.2%) 1	Chi2(2) = 1.525 *p* = 0.467 CC = 0.045
		Girls n (%) CTR	362 (90.7%) −0.2	32 (8%) 0.7	5 (1.3%) −1.0	

CTR: corrected typified residues.

## Data Availability

The data that support the findings of this study are available from the corresponding author upon reasonable request.
